# The Breadth, but Not the Magnitude, of Circulating Memory B Cell Responses to *P. falciparum* Increases with Age/Exposure in an Area of Low Transmission

**DOI:** 10.1371/journal.pone.0025582

**Published:** 2011-10-04

**Authors:** Sarah I. Nogaro, Julius C. Hafalla, Brigitte Walther, Edmond J. Remarque, Kevin K. A. Tetteh, David J. Conway, Eleanor M. Riley, Michael Walther

**Affiliations:** 1 Medical Research Council Laboratories, Fajara, Banjul, The Gambia; 2 Department of Immunology and Infection, Faculty of Infectious and Tropical Diseases, London School of Hygiene and Tropical Medicine, London, United Kingdom; 3 Department of Parasitology, Biomedical Primate Research Centre, GJ Rijswijk, The Netherlands; 4 Department of Pathogen Molecular Biology, Faculty of Infectious and Tropical Diseases, London School of Hygiene and Tropical Medicine, London, United Kingdom; Agency for Science, Technology and Research - Singapore Immunology Network, Singapore

## Abstract

**Background:**

Malaria caused by *Plasmodium falciparum* remains a major cause of death in sub-Saharan Africa. Immunity against symptoms of malaria requires repeated exposure, suggesting either that the parasite is poorly immunogenic or that the development of effective immune responses to malaria may be impaired.

**Methods:**

We carried out two age-stratified cross-sectional surveys of anti-malarial humoral immune responses in a Gambian village where *P. falciparum* malaria transmission is low and sporadic. Circulating antibodies and memory B cells (MBC) to four malarial antigens were measured using ELISA and cultured B cell ELISpot.

**Findings and Conclusions:**

The proportion of individuals with malaria-specific MBC and antibodies, and the average number of antigens recognised by each individual, increased with age but the magnitude of these responses did not. Malaria-specific antibody levels did not correlate with either the prevalence or median number of MBC, indicating that these two assays are measuring different aspects of the humoral immune response. Among those with immunological evidence of malaria exposure (defined as a positive response to at least one malarial antigen either by ELISA or ELISPOT), the median number of malaria-specific MBC was similar to median numbers of diphtheria-specific MBC, suggesting that the circulating memory cell pool for malaria antigens is of similar size to that for other antigens.

## Introduction

The immune system's ability to mount an accelerated humoral immune response upon repeated encounter of the same pathogen allows for rapid reduction in disease severity or even complete sterile immunity [1]. However, in the case of malaria, sterile immunity that would prevent re-infection is rare; clinical immunity is thought to be species and strain-specific and repeated infections are required to develop immune responses specific for the prevalent antigenic types in the area of residence [2]. The rapidity with which effective immunity is acquired thus depends on the rate of transmission [3].

The immune control of malaria infection is multi-factorial, and there is growing consensus that the synergistic action of antibodies (Ab) and cell mediated effector mechanisms is required for both anti-parasitic as well as clinical immunity [2], [4]. The paramount importance of Ab in clearing parasitized red blood cells and reducing clinical symptoms was highlighted several decades ago, by passive immunoglobulin (Ig) transfer experiments [5], [6]. A number of studies have subsequently demonstrated an association between protection from clinical symptoms of uncomplicated disease and levels of anti-malarial Ab [7]. However, the frequency of repeated malaria infections in some children in highly endemic areas, together with anecdotal accounts of apparent loss of immunity in the absence of continuing exposure and experimental evidence of dysfunctional T cell responses, has raised legitimate questions regarding the duration of immune memory to malaria [3], [8], [9].

Longitudinal studies showing that titres of many anti-malarial Ab decline rapidly in children once parasitaemia is cleared after acute infection [8], [10], [11], [12], [13] have contributed to the belief that humoral memory to malaria may be defective. On the other hand, there is a growing body of evidence to indicate that Ab responses become increasingly stable with increasing age [12] and can be long-lived in adults [14], [15], [16], [17]. Technological advances - particularly the development of the B cell ELISpot assay to quantify antibody secreting cells (ASC) as a surrogate of circulating memory B cells (MBC) [1], [18], [19] – are now allowing the cellular basis of humoral immune memory in malaria to be investigated. In this assay, peripheral blood mononuclear cells (PBMC) are stimulated with a cocktail of B cell mitogens in order to stimulate the differentiation of MBC into Ab producing plasma cells (PC) [1] and the secreted Ig is detected by enzyme immunoassay. Using an early version of this assay, Dorfman et al. [20], attempted to identify malaria-specific MBC among PBMC sampled from malaria-exposed Kenyan children but only very few cells were detected [20]. Recent refinements of the technique [21], have enhanced its sensitivity allowing detection of malaria-specific MBC in children from a high transmission area in Mali [22]. Findings from that study demonstrated that accumulation of MBC was both gradual and occurred in a stepwise fashion over many years of repeated exposure [22]. Using a similar method, malaria-specific MBC were also detected in adults from a low endemicity setting in Thailand [15] and these MBC were found to persist - in the absence of re-exposure - for more than 7 years [15]. Despite the enhanced sensitivity of the current ELISpot assay, in all of these studies antigen-specific MBCs could not be detected in a significant proportion of seropositive individuals. Whether this reflects a real absence of MBC or insufficient sensitivity of the assay is currently not clear.

In this study, in an area in The Gambia where malaria transmission has declined substantially over the last decade [23], [24], to unprecedentedly low prevalence [25], we find that the magnitude of the malaria-specific memory B cell response in children is very similar to that in older individuals and that, in all age groups, malaria-specific MBC responses are of similar magnitude to vaccine antigen-specific responses. However, the prevalence of malaria-specific humoral responses and the breadth of the response (as judged by the number of antigens recognised by an individual) increases with age indicating that acquisition of humoral immunity requires repeated exposure to malaria infection.

## Results

### Study procedures and baseline characteristics of the study cohort

118 healthy volunteers from Brefet, Foni District, The Gambia, were selected using age-stratified randomisation into six different age categories (Table S1). Venous blood samples were collected as part of a cross-sectional survey carried out at the end of the dry season (May–June 2009) over a six-week period. At the time of sample collections, study participants were healthy and afebrile. Using qualitative *P.falciparum* PCR, one individual in each of the 1–4, 15–24 and 25–39 year age groups and three individuals in the 10–14 year age group carried parasites, none of which were detected by slide microscopy.

Stool analysis to assess carriage of intestinal helminths and pathogenic gut parasites was carried out on 46 samples, evenly spread across the different age groups. Two (4.3%) had *Giardia lamblia* cysts (individuals stemming from 0–4 and >39 years age groups) and one (2.2%) had hookworm ova (13 years old).

Morbidity surveillance was performed during the transmission season and study participants suspecting they had malaria, or experiencing symptoms compatible with malaria were asked to report to the village health worker who performed a rapid diagnostic test (RDT). Out of eight participants presenting with symptoms, only one had a positive RDT.

At the end of the transmission season, 8 individuals had shown evidence of exposure based on a≥1.5 fold increase to at least one of the malaria specific Ab tested for (see methods section); one of those individuals presented with clinical symptoms compatible with malaria and tested positive by RDT.

### The proportion of individuals with malaria specific Ab increases with age

**Figure 1:**
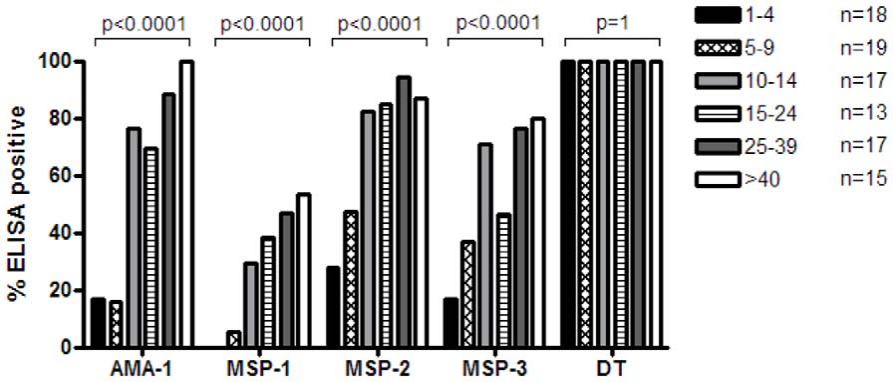
The proportion of individuals with malaria-specific plasma antibodies increases with age. The percentage of individuals having Ab levels above the cut-off for AMA-1, MSP-1_19_, MSP-2, MSP-3 or DT are shown for each age group. Data are for samples collected in May–June 2009, prior to the annual malaria transmission season. N is equal to the number of samples available per age group. P values indicate the result for the Chi-squared test for trend.

Samples collected in May 2009 from Brefet were assayed for Ab specific to MSP-1_19_, MSP-2, MSP-3, AMA-1 and diphtheria toxoid (DT), and the percentages of individuals having Ab to the different Ag were plotted for each age group (Figure 1). The geometric mean anti-diphtheria IgG concentration of the study population was 26.9 IU/ml (CI 95%:22.2 to 32.6) with all study participants having >1.4 IU/ml. Since antibody titres >0.1 IU/ml are regarded by the test manufacturer as a sign of “good immunity”, all participants were classified as responders with regard to anti-diphtheria Ab. For all four malarial Ags the proportion of individuals having malaria-specific Ab increased significantly with age (Chi square test for trend for all Ags: p<0.0001). AMA-1 was the only Ag for which by the age of ≥40 years, all individuals had Abs, confirming that AMA-1 is amongst the most immunogenic of the malarial Ags [16], [26]. In comparison, for MSP-1_19_, considered to be less immunogenic [16], [27], [28], the increase with age was less pronounced with Ab prevalence reaching 60% amongst adults aged ≥40 years.

### Inter-individual variation in the frequency of circulating MBC

**Figure 2:**
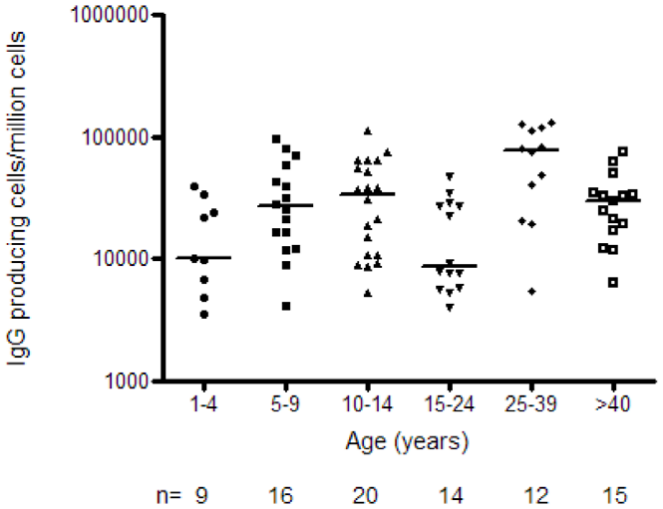
Accumulation of MBC with age. The total number of MBC/million PBMC (as measured by their conversion into IgG producing cells after culture) were counted for each individual using the B cell ELISpot technique, and are shown according to age group. The horizontal lines show the median values for each age group. N equals the number of tests that were performed per age group.

Surprisingly little is known about how the total population of circulating MBCs - as measured by their conversion into IgG ASC in the B cell ELISpot assay - develops with increasing age and, thus, cumulative Ag experience. We therefore plotted the number of ASC per million cultured PBMC for each age group and examined the data for any change in ASC numbers with increasing age (Figure 2). Assuming that the number of ASC detected after in vitro culture is a reliable reflection of the precursor frequency of circulating MBCs, we find considerable inter-individual variation in the frequency of MBC in all age groups (Kruskal Wallis test: p = 0.0024). Although the 1–4 year olds had significantly lower frequencies of MBC than the 25–39 year olds (p<0.05, Dunn's post test), logistic regression across all age groups failed to detect a significant increase with age (p = 0.086); one reason for this may be the anomalous and unexplained drop in total MBC numbers in the 15–24 year age group. However, when data for children above 15 years of age were pooled and the 10–14 year old age group was used as the baseline group, the model suggests that the MBC frequency in 10–14 year olds may be higher than in 1–4 year olds (p = 0.051) but that there is no difference in MBC frequency between 10–14 year olds and those aged 15 years or more (p = 0.802).

### The proportion of individuals with malaria-specific MBC increases with age

Previous studies that have looked at the frequency of malaria Ag-specific MBC measured by ELISpot have reported their results either as the number of malaria-specific spots per million PBMC seeded into the well (MBC/PBMC) [20], [22], or the proportion of all IgG ASC (%MBC) that are malaria antigen-specific [15], [29], [30]. The considerable inter-individual variation we observed in overall MBC frequencies and the possibility that the population of MBC might increase during childhood, clearly indicates that the choice of the denominator may affect the interpretation of the data. In this study, we therefore analysed the ELISpot data using both methods of calculation. Unfortunately, due to a temporary shortage of IgG detection reagents, we were only able to enumerate the total number of IgG producing cells (and therefore calculate the % MBC) for 86 of the 118 participants.

**Figure 3:**
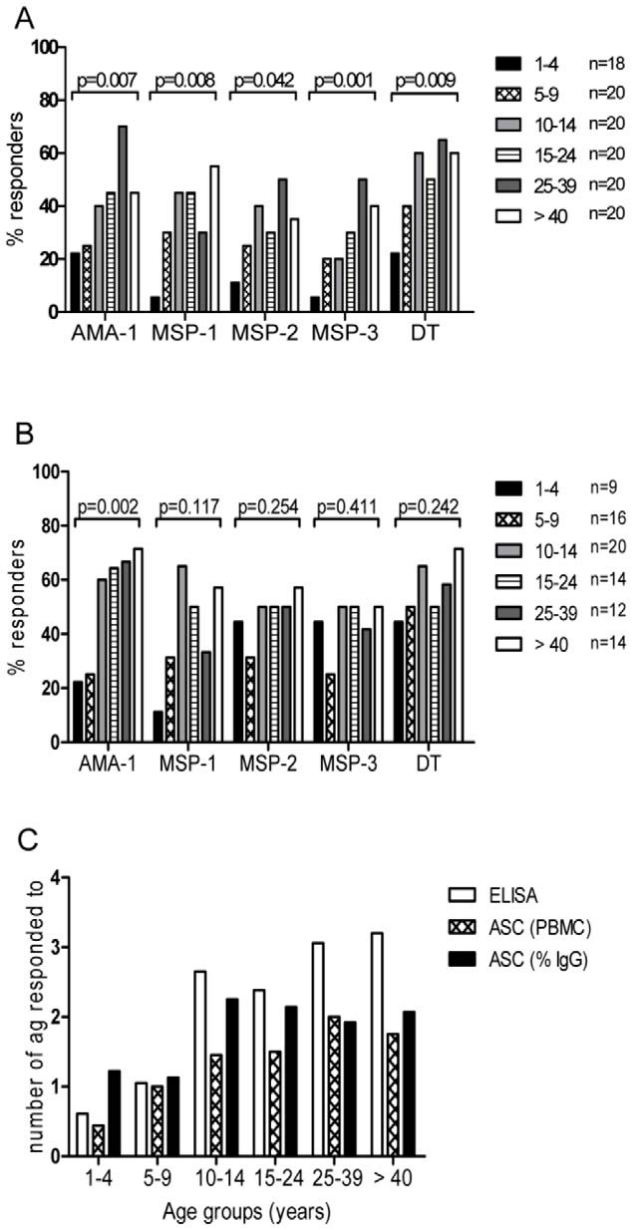
Prevalence and antigenic breadth of the malaria-specific MBC response increases with age. The percentage of individuals in each age group with detectable MBC specific for the different malaria Ags and for DT are shown as (A) number of MBC/PBMC, and as (B) % of all MBC. P-values for a Chi-squared test for trend are shown. N equals the number of samples tested per age group. The average number of Ags for which individuals had specific Ab (open bars) or MBC (hatched bars = MBC/PBMC; black bars = %ASC) are shown, by age group, in (C). For all three parameters, the Chi-squared test for trends indicated a significant increase with age (p<0.0001 for Ab and MBC/PBMC, and p = 0.0083 for %MBC). Positive responses to individual antigens were defined as described in methods.

When the data were expressed as MBC/PBMC, the proportion of individuals with malaria specific MBC above the cut-off (hereafter referred to as responders) increased significantly with age for all Ags (Figure 3A). Responder status was defined as malaria Ag-specific MBC frequencies above the upper limit of the 99% CI of the median MBC/PBMC frequency for the negative control Keyhole Limpet Hemocyanin (KLH), being 1.88 spots/million PBMC. A similar trend was observed when data were expressed as % MBC (Figure 3B; here responder status was defined as values above the upper limit of the 99% CI of the median % MBC determined for KLH, being 0.004%), but, apart from AMA-1, the trends were not statistically significant. Importantly, the two methods of analysis showed a substantial level of agreement with regards to identifying individual responders, with *kappa* values ranging from 0.55 (MSP-2), 0.64 (MSP-1_19_), 0.72 (DT), 0.78 (AMA-1), to 0.81 (MSP-3).

### The breadth of the malaria-specific immune response increases with age

To assess how the breadth of the immune response to malaria Ags develops with age, the average number of malaria Ags recognised by each individual's plasma or cells was stratified by age. As shown in Figure 3C, the number of Ags against which specific plasma IgG or MBC were detected increases significantly with age (Chi-square test for trend p<0.0001 for ELISA and MBC/PBMC; and p = 0.0083 for %MBC). Interestingly, from the age of 10 years, the repertoire of Ags recognised by plasma IgG was significantly broader than the repertoire recognized by MBC (expressed as MBC/PBMC; p value for Chi square for each age strata: <0.012). When the same comparison was carried out between ELISA data and ELISpot data expressed as %MBC, the repertoire of Ags recognized by plasma IgG became significantly broader from 25 years onwards (p value for Chi square for each age strata: <0.0015).

### Lack of correlation between plasma IgG responses and MBCs detectable by ELISpot

**Figure 4:**
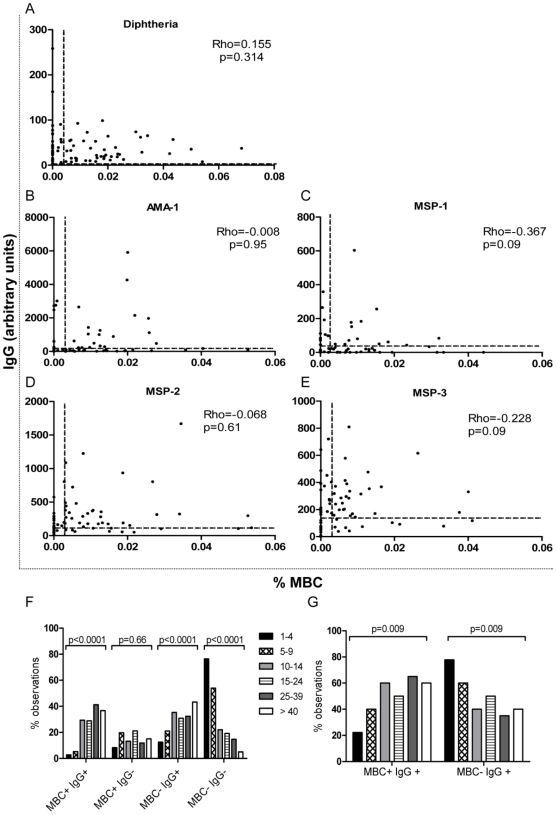
Plasma Ab levels do not correlate with MBC numbers. A–E: correlation plots for Ab versus % MBC. The cut-off values for defining responders are shown by dotted lines parallel to each axis. The degree of correlation was assessed by calculating Spearman's correlation coefficient for data points where one or both values were above the cut-off. F–G: Each individual's response to each Ag were classified as: MBC+ IgG+ (that is, a response in both the ELISpot and the ELISA was seen), MBC+ IgG− (ELISpot response but no Ab), MBC− IgG+ (no ELISpot response but Ab) or MBC− IgG− (no response in either of the tests). The frequency with which each category occurred in each age group was then expressed as a proportion and is plotted for F) malaria Ag and G) DT. Responder status for MBC was determined using MBC/PBMC. P test for trend values are indicated.

IgG measured in plasma by ELISA is an indicator of the presence of antibody-secreting effector cells (plasma cells) in peripheral tissues or bone marrow whereas ASC detected by cultured ELISpot assay indicate the presence of circulating MBC. To explore the relationship between these two very different measures of the humoral immune response, IgG concentrations were plotted against MBC numbers for all subjects for each Ag (Figure 4A–E) and a correlation coefficient was calculated using data points for which one or both variables were above the respective cut-offs. No significant positive correlation was detected between MBC and Ab for any of the Ags tested, regardless whether ELISpot results were expressed as %MBC (Figure 4) or as MBC/PBMC (data not shown).

To explore this data further, we classified each individual's responses to all 4 malaria Ags into the following categories: MBC+ IgG+ (that is, a response in both the ELISpot and the ELISA was seen), MBC+ IgG− (ELISpot response but no Ab), MBC− IgG+ (no ELISpot response but Ab) or MBC− IgG− (no response in either of the tests). The frequency with which each category occurred in each age group was then expressed as a proportion and is plotted in Figure 4F for all the malaria Ags combined and Figure 4G for DT, using MBC/PBMC for the ELISpot data.

The proportion of responses to malaria Ags categorized as MBC+ IgG+ increases with age, while the proportion of responses classified as MBC− IgG− decreases (Chi squared test for trend, p<0.0001 for both), which presumably reflects cumulative exposure to malaria over time. Interestingly however, the proportion of responses classified as MBC− IgG+ also increased with increasing age (Chi squared test for trend: <0.0001) suggesting that antibody secreting plasma cells persist even though the frequency of circulating MBC remains below the threshold of detection.

Similar analysis was performed for responses to DT (Figure 4G). Since all participants had anti-diphtheria Ab, only two categories (MBC+ IgG+ and MBC− IgG+) existed. With increasing age, significantly more responses were classified as MBC+ IgG+ (Chi squared test for trend: 0.009). Since diphtheria vaccination is only given during childhood and natural exposure to diphtheria is now extremely rare in The Gambia [31], we speculate that this change reflects either age-dependent qualitative changes in the immune response or recurrent bystander B cell activation (driving DT-specific MBC into periodic clonal expansion) rather than repeated exposure to diphtheria Ag.

The magnitude of MBC responses expressed as % MBC were only poorly correlated with numbers of MBC/PBMC (Table 1), reflecting the considerable inter-individual variation in total MBC numbers.

**Table 1:** Correlation between the two methods of calculating Ag-specific MBC.

	n	r^2^	p
AMA-1	33	0.297	0.090
MSP-1_19_	30	0.237	0.207
MSP-2	29	0.462	0.012
MSP-3	22	0.135	0.550
DT	44	0.288	0.058

The magnitude of the MBC response, expressed either as MBC/PBMC or % MBC was assessed for correlation. n: number of pairs tested for each Ag; r^2^: Spearman correlation coefficient.

### Similar frequencies of malaria-specific and diphtheria-specific MBCs

**Figure 5:**
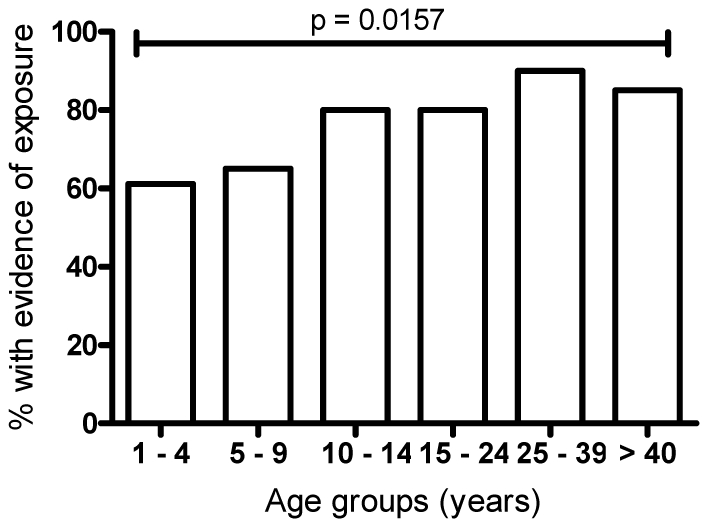
Evidence of malaria exposure in Brefet increases with age. The percentage of individuals showing a response above the respective cut off to at least one of the malarial antigens tested either in the ELISA or the ELISpot assays is shown, stratified according to age. Chi-squared test for trends indicated a significant increase with age (p = 0.0157).

**Figure 6:**
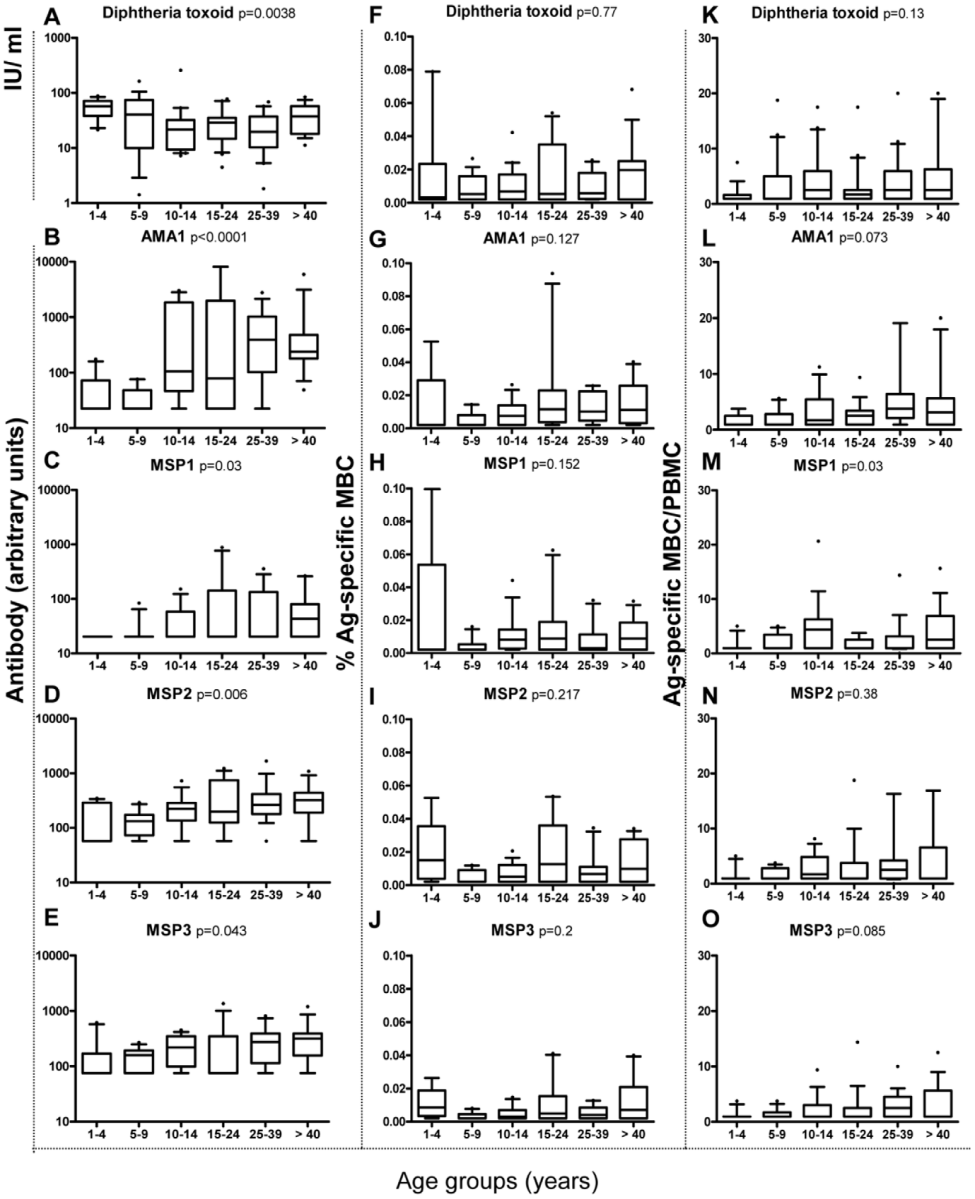
The magnitude of the malaria antigen-specific MBC response does not increase with age. The magnitude of Ag-specific serum IgG (A–E) and of Ag-specific MBC responses [expressed as %MBC (F–J) or expressed as MBC/PBMC (K–O)] are shown for individuals who showed evidence of previous malaria exposure (as defined in Figure 5). Box plots indicate the 25^th^, 50^st^ and 75^th^ percentile, with whiskers representing the 10th and 90th percentiles. Outliers are denoted by a spot. P values are given for Kruskal-Wallis tests.

We were interested to explore i) whether the magnitude of the malaria-specific humoral and B cell ELISpot responses increases with age, and ii) whether the magnitude of malaria specific B cell ELISpot responses differs from DT specific responses. This requires defining each participant's prior exposure to the antigens. Based on the 100% prevalence of IgG against diphtheria in the study group, it can be assumed that every participant has encountered diphtheria antigen in the past. Determining prior exposure to malaria is less straightforward, especially in this community where the very low levels of transmission over the last 10 years mean that the younger individuals may have had very few (if any) previous malaria infections. Extensive polymorphism of malarial blood stage antigens means that even among exposed individuals, prior exposure to the precise antigenic variants used in our assays cannot be assumed. Also, different individuals may respond differently to a given antigen [14] and, as we have shown, circulating IgG antibody may not be detectable despite the presence of corresponding MBCs, and vice versa. Given the very low levels of malaria transmission in Brefet (23) we believe it is justifiable to consider individuals who are seronegative to all four malaria antigens, and who lack detectable MBC to any of these four antigens, to be malaria unexposed; conversely individuals who have IgG or MBC to one or more malaria antigens were considered to have been exposed. By this definition, 60–80% of children and >90% of adults had evidence of prior malaria exposure (Figure 5). Figure 6 shows the magnitude of malaria specific IgG and MBC responses for all participants considered as malaria exposed across age groups, and diphtheria responses for all participants. Consistent with the high coverage of the childhood immunisation programme in The Gambia [31], anti-diphtheria plasma IgG concentrations declined during childhood from a peak in children aged 1–4 years (Kruskal Wallis test, p = 0.004, with significant p value for post test between 1–4 years and 10–14 years, Figure 6A), but were relatively stable from age 10 years onwards. However, there was no significant difference in diphtheria-specific MBC numbers between different age groups (Figure 6F and 6K). By contrast, and as expected from previous studies, the concentration of all malaria-specific antibodies tested increased with age (Figure 6B–E).

For all the Ags tested, Ag-specific MBC frequencies (expressed as %MBC) did not vary significantly across age groups (Figure 6F–J). With the exception of MSP-1_19_ (Figure 6M), for which a significant decrease in MBC numbers was observed between age groups 10–14 and 15–24 years, the same result was obtained when MBC were expressed as MBC/PBMC (Figure 6K, L, N and O).

**Figure 7:**
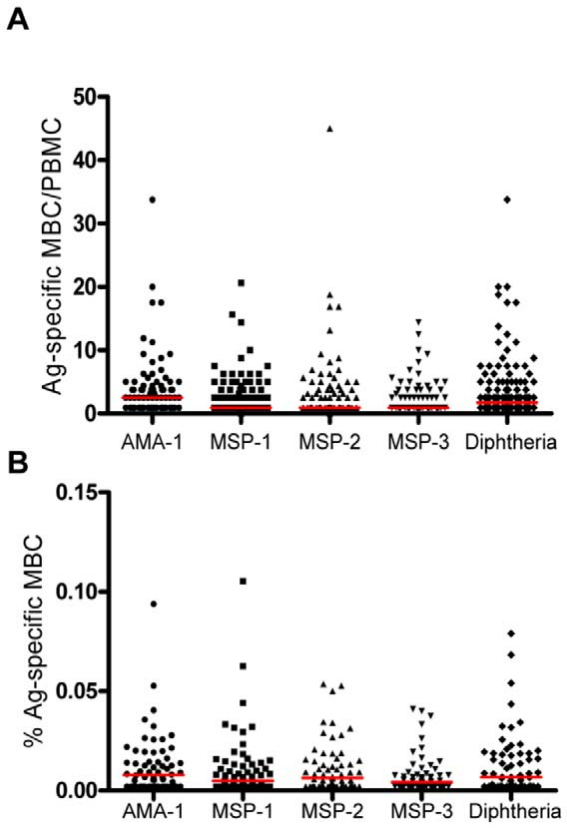
Naturally acquired MBC responses to malaria antigens are similar in magnitude to vaccine-induced MBC responses to DT. Ag-specific ELISpot responses of individuals with immunological evidence of prior malaria exposure are shown and expressed as (A) MBC/PBMC and (B) % MBC, for each Ag. The median for each group is indicated by the horizontal line. The p value (Kruskal Wallis test) for (A) = 0.08, and (B) = 0.178.

Of note, the magnitude of malarial Ag -specific MBC responses amongst those with evidence of previous malaria exposure was not different from that for diphtheria, irrespective of the chosen denominator (Kruskal Wallis test p = 0.08 [MBC/PBMC], or p = 0.178 [%MBC]; Figure 7A, B).

### Effect of recent malaria exposure on malaria-specific plasma IgG antibodies and MBCs

**Figure 8:**
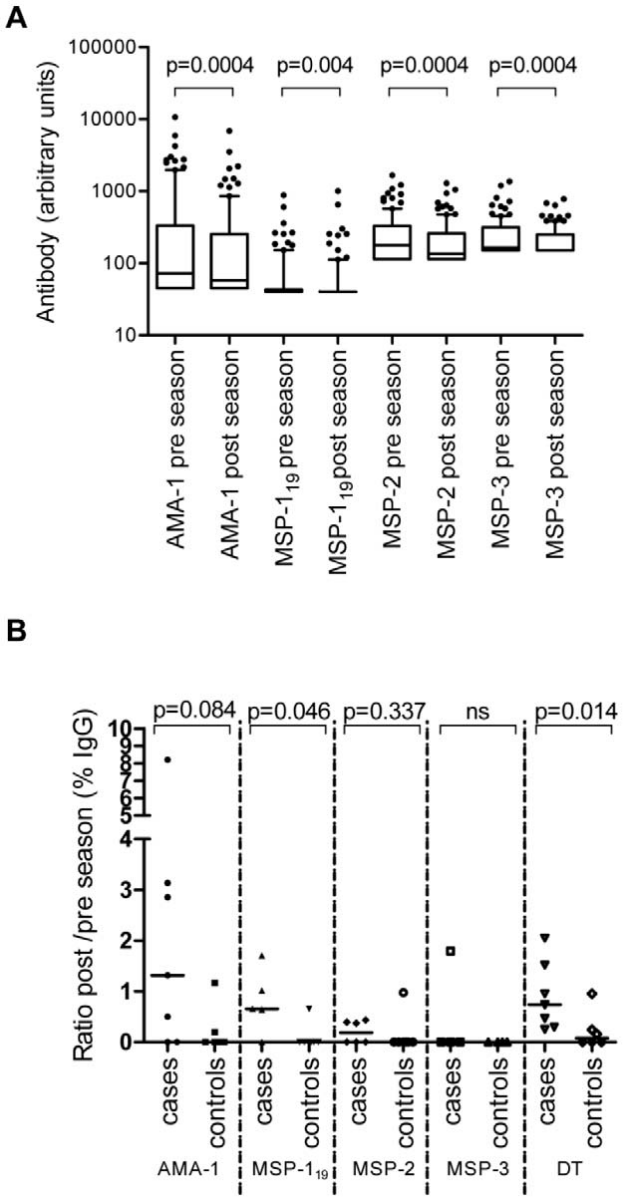
Boosting of circulating MBC numbers by recent malaria infection. (A) Pre- and post-malaria season plasma Ab concentrations to the different malaria Ags are shown for all participants for whom paired samples were available (n = 99), pooled across all age groups. Values below the antigen-specific cut-off (as described in the methods) were given the cut-off value. Box plots indicate the 25^th^, 50^st^ and 75^th^ percentile, with whiskers representing the 10th and 90th percentiles. Outliers are indicated by spots. (B) Comparisons of the ratio of post-season MBC to pre-season MBC for cases (individuals having a>1.5 fold increased Ab concentration post season for at least one of the malarial Ags) and controls. P values are given for the Mann-Whitney test, adjusted for multiple comparisons using the Bonferroni method.

To capture evidence of malaria exposure during the transmission season we performed active morbidity surveillance of our study population from August 2009 till December 2009. Only one symptomatic case with a positive RDT was detected (in a 5 year old child). In addition, we repeated the serological survey in December 2009 and tested these samples together with those collected in May 2009. Taking the study population as a whole we observed small, but significant, decreases in the median concentrations of Abs to AMA-1, MSP-1_19_, MSP-2 and MSP-3 (Figure 8A), confirming the lack of recent re-exposure to malaria in the majority of subjects.

However, 8 (including the 1 RDT positive case) out of 99 participants that were tested showed a≥1.5 fold increase in plasma IgG levels to at least one malaria Ag suggesting that they may have recently experienced a malaria infection; 7 out of 8 of these infections are assumed to have been asymptomatic. PCR carried out at the end of the transmission period detected *P. falciparum* DNA in only one of these 8 subjects. A “rising titre” of serum Ab is widely used as a diagnostic tool and suggests that these 8 individuals were indeed infected with malaria. However, despite the fact that (apart from the one RDT positive case) none of them presented with any signs of illness during the study period, we cannot entirely rule out that the increased malaria Ab titres may be the result of bystander activation of malaria-specific B cells by another asymptomatic infection.

Nevertheless, when we compared the change in frequency (pre- and post-malaria season) of malaria-specific MBCs among the 8 subjects who had evidence of a recent malaria infection (cases) with the change in MBC frequency in 8 age-matched controls with no evidence of recent infection, the post/pre-season ratio for malaria-specific MBCs (calculated as a % of all MBC) tended to be higher in cases than in controls (Figure 8B). While the low numbers of cases precludes the drawing of any firm conclusions, this analysis does suggest that MBC numbers can be boosted in the same manner as serum IgG concentrations. Interestingly, the post/pre-season ratio for diphtheria-specific MBCs was also significantly higher in cases than in controls, suggesting that recent infection may have resulted in polyclonal, antigen-independent, “bystander” activation of diphtheria-specific MBCs.

### Atypical memory B cells

**Figure 9:**
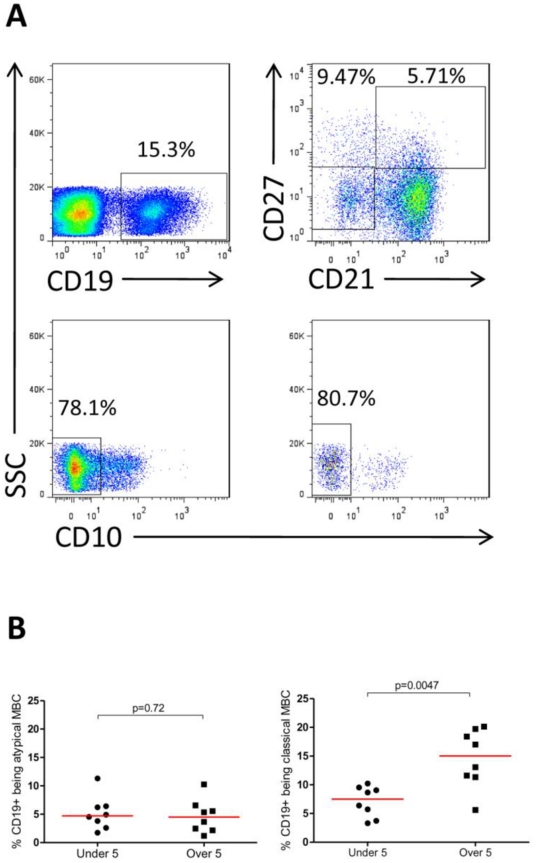
Atypical and classical memory B cells in children 2 months after a malaria episode. AMB were defined as being CD19^+^CD21^−^CD27^−^CD10^−^, classical MBC were defined as CD19^+^CD21^+^CD27^+^ [21]. The gating strategy is shown in (A). Viable PBMC were gated using FSC/SSC (not shown) and then displayed according to CD19^+^ expression. The CD19^+^ population was displayed according to their expression of CD21 and CD27. To identify atypical MBCs, CD10^−^ cells were subsequently selected from CD21^−^CD27^−^ cells, using a CD10 gate defined on the entire PBMC population. (B) Percentages of CD19^+^ cells expressing the atypical and classical MBC phenotype are shown for children <5 and >5 years of age. P-values are given for the Mann-Whitney test.

A recent study reported an increased frequency of dysfunctional and exhausted MBCs (designated atypical memory B cells, AMB) in HIV-infected patients compared to healthy individuals [32]. B cells with a similar surface phenotype (CD19^+^ CD27^−^ CD21^−^ and CD10^−^) have also been reported to be more prevalent in adults from an area with high malaria transmission (Mali) than in malaria-naïve volunteers (from the USA), and their proportion was higher among asymptomatic parasite carriers than among non-parasitized individuals [21]. This prompted us to examine the frequency of AMB with this phenotype in 16 children aged 1–15 years, who were followed up as part of an ongoing malaria case-control study [33], [34]. All children had a confirmed episode of malaria 2 months previously, but were parasite free at the time of blood collection, and live in an area of low malaria endemicity at the Gambian coast [24], [25]. The median proportion of B cells expressing the AMB phenotype (CD19^+^ CD27^−^ CD21^−^ and CD10^−^; Figure 9A) was 4.71% (CI 95%: 2.48–6.41%) with no significant difference between children below and above the age of 5 years (Figure 9B). This compares to AMB frequencies of 15.5% in Malian adults, 9.8% in Malian children (aged 2–10 years) and 1.6% in U.S. adults [21], supporting the hypothesis that malaria induces AMB in an exposure-dependent manner. Interestingly, the proportion of classical MBCs (CD19^+^ CD27^+^ CD21^+^) amongst all B cells was twice as high in children aged more than 5 years than in children aged less than 5 years (Figure 9B), consistent with the age-related increase in the repertoire of antigens recognised by the MBC population.

## Discussion

The advent of methods to enumerate circulating MBC [18] has enabled researchers to begin to explore the induction and maintenance of humoral immune responses to malaria in humans [15], [20], [22]. While the B cell ELISpot may still benefit from further improvements to its sensitivity, the assay is sensitive enough to detect durable MBC responses to both *P.falciparum* and *P.vivax* in infrequently exposed Thai adults [15] and, in a recent report from a rural village in Mali where people are exposed to ∼50 infective mosquito bites per month during the peak of the transmission season, to demonstrate that the *P. falciparum*-specific MBC compartment expands in a step-wise fashion with increasing age and/or repeated malaria infections [22]. Using a similar assay, we have now described the acquisition of humoral immunity to four malaria blood stage antigens (selected on the basis of their immunogenicity and potential functional relevance) in a rural community in The Gambia where malaria transmission has declined sharply in the last 15 years [23], [35] and where only ∼8% of study subjects appear to have been exposed to malaria infection in the 2009 rainy season. The absence of anti-MSP-1_19_ specific IgG in children <10 years in December 2009 [23], the high coverage with insecticide treated nets (ITNs), and the virtual absence of clinical infections in the study cohort all support the assumption that malaria transmission in this community has been extremely low for several years.

Rather surprisingly, given the huge differences in malaria endemicity between the two sites, the age-specific prevalence of both Ab and MBC in The Gambia was remarkably similar to the prevalence reported in Mali [22]. Although there are some slight differences in methodology between the studies (age grouping, definition of cut off values, use of fresh or cryopreserved cells) the similarity in the results is striking. Given the findings of Weiss et al. [22] that acute malaria infections lead to only transient expansion of the malaria-specific MBC pool and that the subsequent contraction of the MBC pool results in very inefficient accumulation of humoral immunity, and the findings of Wipasa et al. [15] of very long-lived B cell memory in areas of exceptionally low transmission, it may be that relatively infrequent exposure to malaria is as effective (or more effective) at inducing long-lived humoral immunity than is persistent re-infection. If so, we perhaps need to consider the possibility that repeated exposure to antigen gradually drives MBC to differentiate into tissue resident cells (either ASCs or non-circulating memory cells) or – possibly – causes clonal exhaustion. On the other hand, the clear increase that we observed in the number of Ags recognised with increasing age indicates that the antigenic breadth (and likely, therefore, the functional relevance) of the B cell response requires at least some degree of repeated exposure to malaria. The underlying biology of these observations clearly deserves further investigation.

A caveat to these interpretations, however, is that the similarity of MBC responses in the different studies may reflect some limitation of the assay system rather than a true biological phenomenon. For example, we find for all Ags, including diphtheria, that although the prevalence of responses varies, the magnitude of the MBC responses (in those classified as exposed) is similar in all age groups. One potential explanation that deserves investigation is that, even in individuals with long-lived humoral immune memory, the maximum precursor frequency of circulating MBCs for any individual Ag is in the order of 1 MBC per million PBMCs and that the spot frequency of 5–10 spots per million PBMC simply reflects the differentiation of these very few MBCs during the 6 days of mitogenic stimulation into MBC. In other words, it may be necessary to seed ELISpot cultures with many more PBMCs in order to detect the true frequency and prevalence of circulating Ag-specific MBCs. Nevertheless, the similar magnitude of the MBC response to malaria Ags and to DT, a protein of fairly similar length to the malaria Ags used, implies that mounting a B cell response to malaria Ags is not necessarily any more difficult than mounting a B cell response to other Ags. Although it is possible that numbers of DT-specific MBC may have been much higher immediately after vaccination in infancy, the lack of any detectable decline in DT-specific MBC frequency with increasing age tends to argue against this possibility.

Another caveat of the B cell ELISpot assay is that we do not know whether the stimulation medium differentiates MBC into ASC at a fixed ratio or whether this is a true reflection of what happens *in vivo* upon encounter with an Ag. Moreover, for different types of Ags the efficiency of the differentiation of MBC into ASC may be different. A direct comparison of MBC frequencies derived from the ELISpot assay with MBC precursor frequencies derived from limiting dilution assays or from antigen-specific flow cytometry (using chromophore-labled antigen) is urgently needed to validate the accuracy and sensitivity of the B cell ELISpot assay.

Related to this is the question of whether B cell ELISpot data should be presented as the proportion of all MBCs that are specific for the Ag of interest (% MBC) [15], [29], [30] or as the number of MBCs among all the PBMCs seeded into the plate (MBC/PBMC) [20], [22]. Our data indicate that for a given number of PBMC, there is considerable inter-individual variation in the total number of MBC. Further, the total number of MBC may increase with age during childhood, presumably as a result of exposure to an ever-increasing number of Ags. Indeed, the total number of MBCs detected in the ELISpot assay tended to be higher in slightly older children than in very young children and, by flow cytometry, the proportion of all B cells that are classical MBCs was also higher in older children than in younger children. Although we could not detect any further expansion of total MBC numbers from adolescence into adulthood, suggesting that MBC numbers may reach an equilibrium in late childhood, our power to detect such a trend was limited by the anomalous drop in MBC numbers in the 15–24 year age group. The reason for this drop is unclear but is highly unlikely to be assay or operator dependent since a random selection of participants across all age groups were tested on each day of the study and the range of values on plates that included low-responding 15–24 years olds did not differ systematically from the ranges on other plates. Given the very low prevalence of HIV infection in this community (∼2%) [36] this drop is unlikely to be related to undiagnosed HIV infection. Further studies in this age group are required. In any event, increasing total MBC numbers are likely to lead to an underestimation of Ag-specific responses when expressed as %MBC. This may be of particular relevance in diseases where the compartment of IgG-producing cells is expanded by means of polyclonal stimulation as has been suggested for malaria [37], [38]. For instance, in the high transmission area in Mali, an extremely marked increase in total MBC numbers with age was observed [22], and reporting results from such a setting as % MBC would further distort the relationship between age and malaria specific memory responses. The number of PBMC per ml of blood is more stable. For this reason, we suspect that MBC/PBMC may better reflect the precursor frequency of malaria specific MBC in the peripheral blood, but further studies are required to be certain that this is the case.

As in most studies of human immune responses, access to the tissues of real interest - in this case spleen and bone marrow - is restricted and observations from studies of PBMC need to be interpreted with caution. In our study, the presence of detectable Abs and MBC did not correlate for any of the Ags tested, and a significant proportion of individuals lacked detectable circulating MBCs despite having significant titres of the corresponding plasma Ab. While this may reflect insufficient sensitivity of the assay, it is also highly plausible that ASCs present in tissues (either as short-lived plasma cells (SLPC) in spleen or as SLPC or long-lived plasma cells (LLPC) in bone marrow) continue to secrete Abs even though the frequency of circulating MBCs has fallen below the lower limit of detection of the ELISpot assay. A similar lack of correlation between MBC and serum Abs was observed in the Thai study [15], where Ag exposure is infrequent, but not in Mali [22], where re-exposure to Ag is very common. These observations, together with the evidence from our comparison of malaria cases and controls in The Gambia, suggest that both Abs and circulating MBC numbers are boosted by re-infection but that, in the absence of boosting, circulating MBC numbers decline to undetectable levels even though tissue resident LLPC continue to secrete Ab. Our observation that the proportion of individuals with Abs but no detectable circulating MBC increases with age - at least for the blood stage malaria Ags tested here - suggests that older people are less likely to have been recently infected, perhaps indicating a degree of pre-erythrocytic immunity in these individuals. However, age-dependent differences in immune responses may also contribute to this effect. Furthermore, due to the marked reduction in malaria endemicity in The Gambia in the last decade, the malaria exposure of children in this community is not only of shorter duration than in adults but is also qualitatively very different from that experienced in childhood by those who are now adults.

B cell mitogenic activity has long been ascribed to *P. falciparum* [37] and has been invoked as the underlying cause of bystander activation of unrelated B cells [38] and of the hypergammaglobulinaemia commonly described in highly malaria endemic areas [39]–[40]. While both the Malian study by Weiss et al. [22] and the present study confirm a bystander effect of acute malaria (boosting tetanus and diphtheria responses respectively), it is far from clear that this is due to the activity of a parasite-derived mitogen as opposed to generalised cytokine-mediated B cell activation.

The functional significance of the relatively high frequencies of cells with the phenotype of so-called “atypical MBCs” in malaria-exposed individuals is not known. One potential explanation is that these cells - which have a substantially shorter life span than classical MBC [32] – have been displaced from bone marrow niches as a result of intermittent polyclonal bystander activation of B cells (after a transient malaria infection, for example) and are destined to die. The observation that such atypical cells are present at much higher frequencies in the high transmission area in Mali [22] than in our low transmission setting or in another area of low transmission in Peru [41] further supports this notion. The fact that the age-related expansion of classical MBCs that we observed is not accompanied by a similar expansion of atypical MBCs in a low endemicity setting tends to support the hypothesis that the frequency of atypical MBCs reflects cumulative malaria exposure and is not just a function of increasing age but, currently, it is not possible to rule out other chronic or recurrent infections (such as helminths, for example), nutritional status or other environmental exposures as contributory causes of this atypical B cell phenotype.

In summary, although the prevalence of individuals with malaria-specific MBCs increases with increasing age (and/or malaria exposure) the magnitude of an individual's malaria specific MBC response is similar in children with minimal prior malaria exposure to adults with a considerably higher cumulative malaria exposure. However, the antigenic repertoire of the B cell response is more limited in children than in adults and only increases with increasing exposure. Since the magnitude of the malaria-specific B cell response is similar to that induced by diphtheria vaccination, we find no evidence that mounting a B cell response to malarial antigens is more difficult than to other antigens. Nevertheless, the possibility remains that the development and/or retention of malaria specific memory B cells is more efficient in areas of low malaria transmission, such as The Gambia or Thailand, than in very highly endemic areas such as Mali. If so, this might be explained either by competition for limited numbers of environmental niches for MBC survival, or by preferential induction of SLPC rather than LLPCs, in frequently infected individuals.

## Materials and Methods

### Ethical Approval

The studies were approved by both the Gambia Government/Medical Research Council (MRC) Joint Ethics Committee and the Ethics Committee at the London School of Hygiene and Tropical Medicine. Participants were enrolled after individual written informed consent was obtained from the participant or their parent/guardian.

### Study site and sample collection

Blood samples from 118 healthy individuals were collected in May–June 2009 (prior to the transmission season) from Brefet, Foni District, The Gambia. Brefet is a rural village situated 55 km inland from the Atlantic coast and 1 km from the river Gambia. In The Gambia, malaria transmission is seasonal, starting in July and ending in December with the peak of transmission being in November [23]. The entomological inoculation rate in the area of Brefet was estimated as 3.24 infective mosquito bites per person per year in 1991 [42], as 0.92 in 2001 [43], and as 0.62 in 2006 [35]. At the time of this survey (in 2009), 82% of beds in Brefet were covered with an insecticide treated bed net.

Villagers were grouped according to age into the following six age categories (1–4, 5–9, 10–14, 15–24, 25–39 and >40 years, respectively) and 20 individuals from each group were randomly selected. To avoid household clustering, all volunteers in each group received consecutive numbers, the total number of villagers per group was noted and out of this figure a random number generator drew 20 numbers to identify 20 individuals per age group. For individuals absent on the day of sampling, a new number was drawn. There was an equal distribution of males and females in each group.

A venous blood sample of 5 to 20 ml was obtained from each participant in accordance with the age-specific guidance provided by the Gambian Government / MRC Joint Ethics Committee. A thick blood film was prepared to test for presence of parasitaemia by slide microscopy, 0.5 ml of blood was collected into EDTA tubes for parasite detection by PCR and the remainder of the sample was collected into heparinised tubes and used for B cell ELISpot and Ab measurement by ELISA. HIV testing was not done on any of the samples, and HIV status was not reported. Adult HIV prevalence in rural areas of The Gambia is estimated to be approx 2% in 2009 [36]; thus undiagnosed HIV infection is unlikely to have markedly affected the immune responses reported here. Stool samples from 38% of randomly selected study participants were also collected to assess worm carriage (Table S1).

A village health worker was present in Brefet throughout the transmission season to diagnose malaria infections by use of RDT (OptiMAL®) for malaria. Study participants had been asked to report to the village health centre if they suspected they had malaria. Anti-malarial treatment was only given if the rapid diagnostic test was positive.

At the end of the transmission season (December 2009), a finger prick sample from each study participant was obtained to assess i) anti-malarial Ab levels, and ii) parasitaemia levels by PCR. Samples from 20 individuals were missing (18 had travelled away from the village and 2 had withdrawn their consent). A week later, a further venous sample for B cell ELISpot was requested from those individuals who showed a≥1.5 fold increase in Ab concentration for at least 1 Ag in comparison to the value recorded at the start of the malaria season in May and/or for whom a clinical episode of malaria during the last transmission season was confirmed by a positive RDT. Individuals with increased Ab concentrations and/or a clinical episode of malaria are collectively referred to as cases. Age-matched (plus/minus 1 year) controls (study participants without evidence of malaria exposure during the transmission season) were selected from the study cohort for comparison.

For the measurement of AMB, blood collected from children enrolled in an ongoing malaria case-control study based in the coastal area of The Gambia [33], [34] was used.

### Antigens

The MSP-1_19_ and MSP-2 Ags used were glutathione s-transferase (GST) fusion proteins representing amino acids 1631–1726 of the Wellcome allele of MSP-1_19_ [44] and amino acids 22–247 of the Dd2 allele of MSP-2 [45], respectively. MSP-1_19_ is highly conserved among *P. falciparum* isolates with minor sequence variations having a minimal effect on antibody recognition [28]. Parasites belonging to the MSP2-Dd2 family are widely represented amongst parasite isolates in The Gambia [46]. The MSP-3 Ag used is a maltose binding protein (MBP) fusion protein representing amino acids 2–354 of the 3D7 allele of MSP-3 [47]. The AMA-1 Ag is a 6×Histidine tagged protein representing amino acids 22–545 of the 3D7 allele sequence [48], [49]. Although there is almost an infinite array of AMA-1 alleles, differing slightly from each other in sequence and antigenicity, polyclonal antibody responses of individuals living in endemic areas appear to cross-react extensively with heterologous AMA-1 sequences [50]. All the Ags were further diluted in 1×PBS at the appropriate working concentrations (0.002 µg/ml). Purified diphtheria toxoid (DT) (NIBSC, UK), derived by formaldehyde deactivation of diphtheria toxin, a 535 amino acid long polypeptide, was used at 0.002 mg/ml to establish diphtheria vaccine induced responses. KLH (from Calbiochem) was used as a control Ag at 0.002 mg/ml.

### ELISpot assay

PBMC were isolated from the participant's whole blood as described elsewhere [33]. Following a protocol adapted from Crotty et al. [1], [19], 1×10^6^ PBMCs in RPMI containing 10% fetal calf serum, 100 U/ml penicillin, 100 ug/ml streptomycin and 2 mM L-glutamine (all Sigma) were added to each well of a 24-well culture plate, and supplemented with 50 µM β-Mercaptoethanol, 0.5 µg/ml Phytolacca Americana pokeweed mitogen (PWE, Sigma), Staphylococcus *aureus* Cowan (SAC, Sigma), 3 µg/ml CpG-2006 (Eurofins MWG-Operon- 5′TCG TCG TTT TGT CGT TTT GTC GTT 3′) and 25 ng/ml recombinant human IL-10 (R&D Systems) and placed in the incubator (37°C, 5% CO_2_) for six days. On day 5, quadruplicate wells of multiscreen-HA plates (Millipore, MAHAS4510) were coated overnight with either unbiotinylated AffiniPure F(ab′)_2_ fragment donkey anti-human IgG (Jackson ImmunoResearch Laboratories), or with one of the four malarial Ags, DT or KLH, respectively. On day six, 400,000 cultured cells were added to each antigen coated well (2000 cells for IgG coated wells), and incubated for 6 hours (37°C, 5% CO_2_). Cells were then incubated overnight (at 4°C) with biotin-SP-conjugated AffiniPure fragment donkey anti-human IgG (Jackson ImmunoResearch). The next day, strepavidin-AKP (BD Biosciences) followed by detection solution (AP conjugate substrate kit (BioRad)) was added and plates were read using the AID ELISpot reader. All samples in the IgG coated wells yielded more than 1000 spots/million PBMC, a threshold previously defined for a valid assay [22]. The median of the coefficients of variation for the replicates from all subjects and all antigens was 8.03%. Malaria-specific ASC are presented either as the percentage of the average number of spots counted in the IgG coated wells (% MBC), or as spots per million PBMC (MBC/PBMC) seeded onto the ELISpot plate after the 6 day culture.

Only values above the upper limit of the 99% confidence interval of the median obtained for the negative control KLH (0.004% or more than 1.88 spots/million PBMC) were considered as positive responses.

### ELISA for serum antibodies to malaria antigens

Concentrations of IgG binding to MSP-1_19_, MSP-2, MSP-3 and AMA-1 were measured by ELISA, using the method described elsewhere [12], [51]. Ab levels to the different malaria antigens tested were compared to a pool of sera samples collected in Brefet in 2008, and expressed as arbitrary units. Results obtained with a 50 fold dilution of the pooled sample were defined as 20 arbitrary units.

A positive response was defined by a value above the upper limit of the 99% confidence interval obtained for this Ag with a pool of plasma samples obtained from 20 malaria naïve tourists from Europe. Only individuals which were above the cut-off value were termed responders.

### Diphtheria toxoid IgG ELISA

Ab levels to DT were quantified using a commercial kit (MP Biomedicals Diphtheria Toxoid IgG antibody ELISA kit catalogue number 071-524002) following the manufacturer's instructions. OD values are converted to International Units (IU/ml) by comparison with the standard curve. The manufacturer considers antibody titres >0.1 IU/ml sufficient for protection.

### PCR

DNA was extracted from 150 µl of EDTA blood using the X-tractor GeneTM robot, according to the manufacturer's instructions (Corbett Robotics) [35]. PCR amplification of *Plasmodium falciparum* ribosomal DNA was performed as described previously [52]. PCR products were resolved by 2% agarose gel electrophoresis (30 mins, 100 v); gels were stained with ethidium bromide and read by a trans-illuminator (BioRad).

### Flow cytometry analysis

Surface staining of AMB was performed on freshly isolated PBMC. The cells were stained with the following fluorochrome-labelled mouse anti-human antibodies: ECD-anti-CD19 (Beckman Coulter), and APC-anti-CD21, APC-Cy7-anti-CD27, Pe-Cy7-anti-CD10 (all from eBioscience). AMB were identified as follows: CD19^+^CD21^−^CD27^−^CD10^−^. All samples were acquired on a 9-colour CyAn ADP flow cytometer and were analysed by FlowJo software (TreeStar Inc., Ashland, OR, USA).

### Stool analysis

Stool samples were collected into fixative (10% formalin) and processed using the ParasiTrap fecal diagnostic system (Biosepar) and read by the routine microbiology laboratories at the MRC in Fajara, The Gambia.

### Statistical analysis

Data were analysed using GraphPad Prism 5 and STATA 10.1. Chi-squared tests for trend were used to assess whether the percentage of individuals having Ab and MBC changed significantly with age for each Ag as well as for measuring the breadth of the immune response. Kruskal-Wallis tests were used to assess whether Ab concentration or MBC numbers differed significantly between age groups, and to test for differences in MBC numbers for the different Ags. As appropriate, Wilcoxon matched paired tests or Mann-Whitney tests were performed to compare two groups. Where multiple tests were performed on the same individuals for multiple responses to different malarial Ags, p values were adjusted for multiple comparisons using the Bonferoni correction. To assess the agreement between the two read outs used for the ELISpot results (% MBC or MBC/PBMC) in identifying “responders” Kappa statistics were calculated, and the results graded using the classification by Landis & Koch [53]. Correlation of two quantitative variables was assessed by calculating the Spearman's correlation coefficient. A logistic regression model was used to analyse the changes in the frequency of MBC with age.

## Supporting Information

Table S1 Baseline characteristics of the study cohort.
(DOC)
