# Supplementary material for: The Breadth, but Not the Magnitude, of Circulating Memory B Cell Responses to P. falciparum Increases with Age/Exposure in an Area of Low Transmission
Source: PLoS One. 2011 Oct 4;6(10):e25582. doi: 10.1371/journal.pone.0025582 (PMC3186790; doi:10.1371/journal.pone.0025582)
Supplement: Table S1 — Baseline characteristics of the study cohort. (DOC) [file pone.0025582.s001.doc]

**Table S1.. Baseline characteristics of the study cohort.**

|  | Age groups (years) | | | | | |
| --- | --- | --- | --- | --- | --- | --- |
|  | 1-4 | 5-9 | 10-14 | 15-24 | 25-39 | >40 |
| Number of participants recruited | 18 | 20 | 20 | 20 | 20 | 20 |
| Median age (years) | 3.5 | 7.5 | 12.5 | 18.5 | 31.5 | 55 |
| Gender, % female (no) | 50 (9) | 55 (11) | 45 (9) | 50 (10) | 55 (11) | 50 (10) |
| Proportion of parasite positive by slide (no) | 0 | 0 | 0 | 0 | 0 | 0 |
| Proportion of parasite by PCR (no) | 6.25* (1) | 0 (0) | 15 (3) | 5 (1) | 5 (1) | 0 (0) |
| Number of GI helminth infections: no pos/no tested | 1/6 | 0/7 | 1/7 | 0/4 | 0/10 | 1/12 |

**Dry season survey. 118 individuals were included in this survey. * PCR results were available for 16 samples.**
